# Real-time monitoring of single dendritic cell maturation using deep learning-assisted surface-enhanced Raman spectroscopy

**DOI:** 10.7150/thno.100298

**Published:** 2024-10-14

**Authors:** Cai Zhang, Mengling Wang, Houde Wu, Hongmei Gao, Pengyun Wu, Li Guo, Dingbin Liu

**Affiliations:** 1 Department of Radiology, Tianjin Medical University Cancer Institute and Hospital, National Clinical Research Center for Cancer, Tianjin's Clinical Research Center for Cancer, Key Laboratory of Cancer Immunology and Biotherapy, Tianjin 300060, China.; 2 College of Chemistry, Research Center for Analytical Sciences, State Key Laboratory of Medicinal Chemical Biology, Tianjin Key Laboratory of Molecular Recognition and Biosensing, Nankai University, Tianjin 300071, China.; 3 School of Medical Imaging, Tianjin Medical University, Tianjin 300203, China.; 4 Department of Nuclear Medicine, Tianjin Medical University General Hospital, Tianjin, 300052, China.; 5 Department of Intensive Care Unit, Key Laboratory for Critical Care Medicine of the Ministry of Health, Emergency Medicine Research Institute, Tianjin First Center Hospital, Nankai University, Tianjin 300192, China

**Keywords:** SERS, Deep learning, Dendritic Cell, Real-time, Single-Cell

## Abstract

**Background:** Dynamic real-time detection of dendritic cell (DC) maturation is pivotal for accurately predicting immune system activation, assessing vaccine efficacy, and determining the effectiveness of immunotherapy. The heterogeneity of cells underscores the significance of assessing the maturation status of each individual cell, while achieving real-time monitoring of DC maturation at the single-cell level poses significant challenges. Surface-enhanced Raman spectroscopy (SERS) holds great potential for providing specific fingerprinting information of DCs to detect biochemical alterations and evaluate their maturation status.

**Methods:** We developed Au@CpG@PEG nanoparticle as a self-reporting nanovaccine for DC activation and maturation state assessment, utilizing a label-free SERS strategy. Fingerprint vibrational spectra of the biological components in different states of DCs were collected and analyzed using deep learning Convolutional Neural Networks (CNN) algorithms, aiding in the rapid and efficient identification of DC maturation.

**Results:** This approach enables dynamic real-time detection of DC maturation, maintaining accuracy levels above 98.92%.

**Conclusion:** By employing molecular profiling, we revealed that the signal ratio of tryptophan-to-carbohydrate holds potential as a prospective marker for distinguishing the maturation status of DCs.

## Introduction

Dendritic cell (DC), renowned as the most potent antigen-presenting cell, plays a pivotal role in regulating both innate and adaptive immune responses. DC exhibits two distinct functional states: mature and immature 1-3. As DC undergoes maturation, the expression of MHC-II and co-stimulatory molecules increases, accompanied by the secretion of cytokines essential for T-cell activation. Mature DC (MDC) initiates metabolic and gene transcription programs. A hallmark of MDC is the ability to activate antigen-specific naive T-cells, thereby initiating antigen-specific immune responses, which have become a competitive alternative for cancer therapy 4-6. However, the tumor microenvironment can inhibit DC maturation and activation, suppressing their antigen-presenting function and thereby failing to effectively activate T-cells. The maturation state of DC profoundly impacts their immunizing activity *in vivo*. Immature DC (ImDC) can either enhance tolerogenicity or promote pro-tumorigenic responses, while MDC robustly induces anticancer immunity. Clinical research on DC vaccines for various tumors has gained attention due to their strong antigen-presenting activity and T-cell activation properties 7. Consequently, assessing the maturation state of DCs is crucial for evaluating immune system activation, vaccine efficacy, and predicting the effectiveness of immunotherapy.

Distinct combinations of DC phenotypic markers, DC-derived cytokines and chemokines, along with other characterized entities such as exosomes, collectively define the nature and progression of DC maturation 8-11. In comparison to ImDC, MDC exhibits notable differences in receptor expression, cytokine secretion, and nucleic acid profiles. Traditional methods for assessing DC maturation primarily rely on enzyme-linked immunosorbent assay (ELISA) for cytokine detection and flow cytometry for the expression of surface marker analysis 12, 13. However, these methods rely on costly antibodies and involve complex procedures. The heterogeneity of cells highlights the importance of analyzing the maturation status of individual cells. To uncover the complexity of individual cellular activities and accurately predict their effectiveness in triggering immune activation in subsequent processes, novel technologies are crucial for real-time analysis of the maturation status of single-DC.

Raman spectroscopy provides unparalleled fingerprinting capabilities for biomolecules, as the spectra reflect the vibrational and rotational modes of molecules, ensuring the extraction of rich biological insights from cells and tissues 14-23. Raman strategy provides exceptional specificity for biological analysis by capitalizing on the distinctive molecular fingerprints present within complex and intact biological samples 24-26, serving as an ideal tool for studying individual cells 27-32. All cellular components contribute to Raman signals, yielding distinct spectral characteristics. Single-cell Raman spectrum serves as a phenotypic fingerprint encompassing all biomolecules within the cell, holding the potential to distinguish between different cell types and provide insights into the underlying biology. Nonetheless, the weak Raman intensity of biomolecules hampers biomedical research, limiting its biological applications. Fortunately, surface-enhanced Raman spectroscopy (SERS) is a sensitive analytical tool holding the potential to dramatically amplify the Raman intensity with an enhancement factor up to 10^7^-10^14^ due to the electromagnetic field in a nanogap between plasmonic surfaces 33-46.

Label-free SERS technology has been explored for studying single-cell heterogeneity 47, biochemical variances across different cell cycles 48-50, cell death mechanisms, and cell proliferation 51. Therefore, SERS technology has great potential for providing specific fingerprinting information of DCs to detect biochemical changes and assess their maturation status. However, the complex and heterogeneous signal patterns provided by various biomolecules constituting cells play a major role in the recognition of the maturation of DCs. Artificial intelligence techniques such as deep learning have been widely employed to extract distinctive features from SERS spectra, enabling the classification of different types of cells, exosomes, and tissues 52-63. Thus, the collaboration of SERS strategy with deep learning algorithms shows great potential in various biomedical processes and offers an opportunity to identify meaningful patterns in the status of dendritic cells.

**Figure 1:**
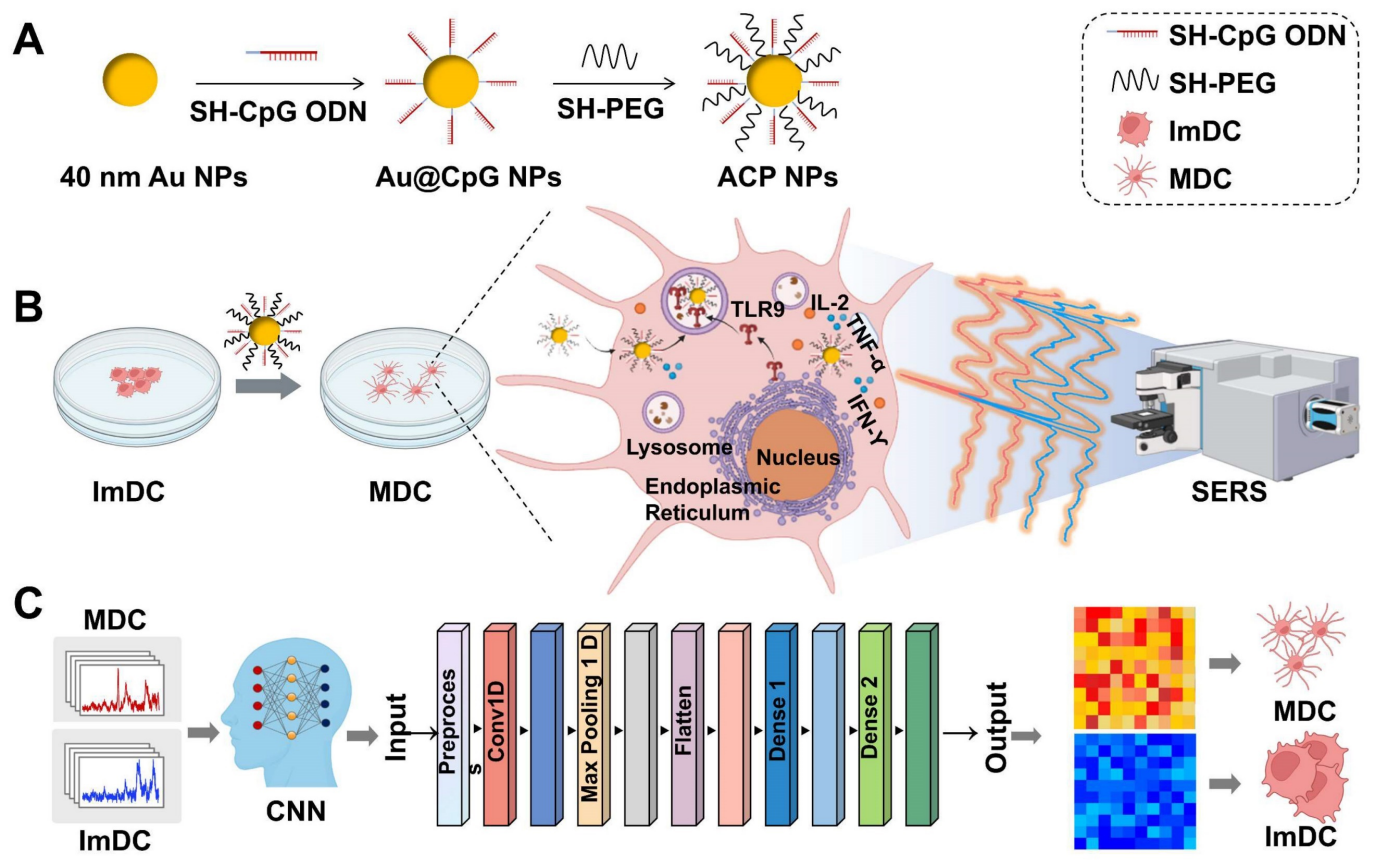
(A) Synthesis of ACP NPs. (B) Collection of spectroscopic data of DCs. (C) The deep learning-based framework for identifying the maturation state of DCs, utilizing SERS spectra from DCs in various states for classification.

Herein, we developed the Au@CpG@PEG nanoparticles (ACP NPs) as the self-reporting nanovaccine for DCs activation and maturation state assessment based on the label-free SERS strategy. The CpG oligodeoxynucleotides (abbreviated as CpG ODNs) are widespread in the genetic sequences of bacteria and viruses, which have been proven to possess the ability to activate DCs through toll-like receptor 9 (TLR9) mediation. The gold nanoparticles (Au NPs) served as both a vehicle for CpG intracellular delivery and a Raman-enhanced substrate due to their excellent enhancement performance and powerful loading efficiency. This dual role ensures the efficiency of cellular uptake, stability of CpG sequences against nuclease degradation, bioactivity of CpG sequences, and significant enhancement of Raman signals from intrinsic cellular components. Fingerprint vibrational spectra of the biological components in different states of DCs were collected and analyzed by Convolutional Neural Network (CNN) algorithms, facilitating rapid and efficient identification of DC maturation (**Figure 1**). Leveraging the Raman spectra self-reporting Au@CpG@PEG nanovaccine in conjunction with deep learning technology enables precise assessment of single-DC maturation status. Moreover, this approach allows real-time monitoring and evaluation during the maturation process of individual DCs, achieving accuracy levels above 98.92%.

## Materials and Methods

**Chemicals and Reagents:** Chloroauric acid and sodium citrate were bought from Sigma-Aldrich. Thiol polyethylene glycol (SH-PEG, MW 2000) was sourced from J&K Scientific. All oligonucleotides were purchased from Sangon Biotech Co., Ltd. (Shanghai) and dissolved in ultrapure water refer to the instruction manual. DMEM, 0.05% trypsin-EDTA and fetal bovine serum were bought from GIBCO.

**Characterization:** UV-vis absorption spectra of the prepared nanoparticles were measured with a UV-3600 plus spectrophotometer (Shimadzu, Japan). Raman spectra were recorded by a Raman microscope (Renishaw) system with a 633 nm laser. Morphology of the nanoparticles was characterized by transmission electron microscopy (TEM, HITACHI HT7700 Exalens). Dynamic light scattering and zeta potentials were measured with a Malvern Zetasizer (Nano seriesnZS, UK).

**Preparation of Au NPs:** 40 nm Au NPs were synthesized using the classical sodium citrate reduction method. Initially, HAuCl_4_ solution (294 μL, 100 mM) was added to 100 mL of ultrapure water in a 250 mL three-neck flask. The solution was then heated to boiling under vigorous stirring. Subsequently, sodium citrate solution (1.5 mL, 10 mg/mL) was quickly added, and the reaction continued for 20 min. The resulting Au NPs solution was slowly stirred until cooled to room temperature.

**Preparation of Au@CpG NPs, Au@Non-CpG NPs, Au@CpG@PEG NPs, Au@Non-CpG @PEG NPs:** Initially, the obtained Au NPs underwent a 20-fold concentration through centrifugation (8000 rpm for 10 min). Next, 110 μL of 100 μM thiol-modified CpG ODNs (or Non-CpG ODNs) were added to 1 mL of the Au NPs solution (2.7 nM) under magnetic stirring. Non-CpG ODNs were used as the negative control for the fabrication of Au@Non-CpG NPs. NaCl solution was dropped wisely added for facilitating the DNA functionalization process of Au NPs. The final concentration of NaCl was maintained at 0.1 M, and 0.01% Tween 20 was added to the reaction mixture for stabilizing the Au NPs. Then the mixture was incubated at room temperature for 24 h under gentle stirring. The obtained solution was centrifuged to remove free CpG ODNs (or Non-CpG ODNs) and NaCl, and then the precipitates were redispersed in 0.5 mL of 0.1% Tween 20 solution. Finally, Au@CpG NPs (or Au@Non-CpG NPs) solution was mixed with SH-PEG (1 μM) and allowed to react for 30 min. SH-PEG was conjugated to the nanoparticle surface to avoid non-specific adsorption and improve probe stability 64. After that, the obtained Au@CpG@PEG (ACP NPs) solution was centrifuged (8000 rpm for 10 min) for 3 times to remove the free SH-PEG. Au@Non-CpG@PEG NPs (abbreviated as ANP NPs) were prepared as the negative control to evaluate the immunostimulatory effects of ACP NPs. The purified ACP NPs (or ANP NPs) solution was stored at 4 °C for further use.

**Cytotoxicity Evaluation of the ACP NPs and ANP NPs:** DC2.4 cells were cultured in a complete DMEM medium composed of 10% FBS, 1% penicillin-streptomycin, glutamine, and β-ME at 37 °C with 5% CO_2_. The classical MTT method was used to investigate the biosafety of the ACP NPs at a cellular level. DC2.4 cells were placed in the 96-well plate with about 10^4^ cells per well and cultured for 24 h at 37 °C. Subsequently, the cell supernatants were replaced by the medium that containing various concentrations of ACP NPs or ANP NPs (0, 0.02, 0.05, 0.1, 0.2 nM), and further cultured for another 24 h. After that, the cell samples were washed with PBS, and cultured with the medium which contained 5 mg/mL MTT for 4 h. Then the cell supernatants were removed. Afterward, 120 μL of dimethyl sulfoxide was added into each well to dissolve the formazan crystal accumulated on the bottom. Ultimately, the absorbance at 490 nm of the cell samples was monitored by a microplate reader.

**Cytokine Detection:** DC2.4 cells were placed in the 96-well plate and cultured overnight. Then Au NPs, ACP NPs or ANP NPs were added to the cells with a final concentration of 0.14 nM and incubated for various time points. Subsequently, supernatants of the cell samples were collected for interleukin-6 (IL-6) and tumor necrosis factor α (TNF-α) detection. The secreted cytokine was monitored by the cytokine-specific ELISA kits. The supernatants of the cell samples without any treatment were set as the control. All the samples were detected three times.

**Flow Cytometry:** DC2.4 cells were seeded in 12-well plates and cultured overnight. ACP or ANP nanoparticles were added to the cells at a final concentration of 0.14 nM and incubated for 2 h. Cell samples without any treatment served as the control. Subsequently, the cells were digested with trypsin and resuspended in PBS, followed by staining with CD80 (FITC-labeled) and CD86 (PE-labeled) antibodies at room temperature for 1 hour. After two washes with PBS, the fluorescence expression levels of the CD80 and CD86 markers were analyzed.

**Raman Detection of the DCs:** Glass slides were placed on the bottom of the 12-well plates, and then DC2.4 cells (1×10^4^ cells/well) were plated onto the glass slides. The cells were cultured at 37°C overnight. ACP NPs or ANP NPs solution (0.14 nM) was added to the cells co-incubated for about 20 h. After washed with PBS for three times the cell samples were fixated with 4% paraformaldehyde and then washed with PBS again. Finally, the Raman spectra of single-cell and multiple cells were recorded by a Raman confocal microscope (633 nm laser), with a power of 5 mW, exposure time of 1 s, and a 63× objective lens. For the acquisition of single-cell spectra, an individual cell was precisely positioned within the scanning area, and SERS spectra were systematically recorded at 1 μm intervals. In the case of multicellular spectral acquisition, multiple cell groups were randomly selected, and spectra were obtained under identical experimental conditions. To detect Raman spectra of living cells, the DC2.4 cells (1×10^4^ cells) were plated in the confocal dish cultured overnight. Then the cells were co-incubated with the ACP NPs or ANP NPs for different time points. After being washed with PBS, the Raman spectra of these living cells were detected by the Raman confocal microscope (633 nm laser), with an exposure time of 0.2 s, a 63× objective lens. The living individual cell was positioned within the scanning area, and SERS spectra were recorded at an interval of 1 μm.

**Deep Learning:** Before starting the artificial intelligence processing, the max-min normalization method was applied to the spectral data, scaling the spectral intensities to a common range, specifically the interval 0-1. To identify the most suitable AI algorithm for spectral discrimination, we assessed the performance and theoretical advantages and disadvantages of 3 representative algorithms including CNN, DNN and MLP. The Raman spectrum data for this study was randomly shuffled and divided into a training set, a validation set, and a test set. The strategy parameters were set as follows: Epochs=50, Batchsize=32, with stochastic gradient descent (SGD) chosen as the optimizer. During each iteration, SGD updates the model parameters using calculated gradients to minimize the loss function. The learning rate was set to 0.01 with a momentum of 0.9. The loss function used was binary cross-entropy. A learning rate scheduler and callbacks were employed to prevent overfitting, with a patience of 10 and a minimum learning rate of 0.00001. The loss value indicates the discrepancy between the model's predictions and the actual results in deep learning. A lower loss value signifies closer alignment between the model's predictions and actual results, indicating better performance. On the other hand, represents the ratio of correctly classified samples to the total number of samples in a classification task, reflecting the overall classification ability of the model across all categories. Additionally, the contribution of individual Raman spectral peaks to the CNN model's classification performance was evaluated.

## Results and Discussion

### Preparation and Characterization of ACP NPs

Realization of the production of ACP NPs was started with the synthesis of 40 nm Au NPs, which according to the classical sodium citrate reduction method 65. Then CpG ODNs and PEG were modified on the surface of Au NPs. The addition of NaCl can increase the ionic strength of the solution, which shield electrostatic repulsion between negatively charged DNA molecules, allowing more DNA to bind closely to the gold nanoparticles 66. Therefore, NaCl solution was added for enabling DNA to gradually stabilize and densely cover the Au NPs surface. Non-CpG ODNs were used as the negative control for the fabrication of Au@Non-CpG NPs 67, 68. The size and morphology of Au NPs and Au@CpG NPs were characterized by TEM (**Figure S1A-B**). The Au NPs size analysis was performed, and the size range was 39.75±0.1 nm **(Figure S2)**. A classic core-shell nanostructure was clearly evident, with the CpG shell measuring about 4 nm in thickness, thus verifying the formation of the Au@CpG NPs. The UV-Vis spectra of Au NPs, Au@CpG NPs and ACP NPs were determined using a UV-3600 plus spectrophotometer. The absorption peak of Au NPs was observed at 528.5 nm. After being coated with CpG or Non-CpG ODNs and PEG, the nanoparticles exhibited redshifted absorption peaks, confirming the successful modification with CpG or Non-CpG ODNs and PEG (**Figure S1C, Figure S3B**). Dynamic light scattering (DLS) was conducted using a Malvern Zetasizer ZS instrument (Malvern Zetasizer 3000HS) to monitor the hydrodynamic diameter of the nanoparticles. As shown in **Figure S1D**, the hydrodynamic sizes of Au NPs, Au@CpG NPs and ACP NPs were approximately 44 nm, 51 nm and 59 nm, respectively. The hydrodynamic diameters of the Au@Non-CpG NPs and ANP NPs were also recorded (**Figure S3C**). The increase in hydrodynamic diameter indicated the successful binding of CpG ODNs (Non-CpG ODNs) and PEG on the surface of Au NPs. The Zeta potential of the nanoparticles was determined, as shown in **Figure S3A.** The surface charge of the Au NPs exhibited a significant negative charge due to the abundant surface presence of sodium citrate. Following CpG or Non-CpG and PEG modification, the negative charge is progressively neutralized, leading to a diminished negative charge.

### Cytotoxicity Evaluation of the ACP NPs

The DC2.4 cell line, derived from murine bone marrow, is extensively used in immunological research to investigate dendritic cell functions such as antigen presentation, cytokine production, and activation mechanisms 69. Therefore, the DC2.4 cell line was selected for the cellular experiments. The cytotoxicity of the ACP NPs and ANP NPs was evaluated by the classical MTT method. DC2.4 cells were cultured in the 96-well plate for 12 h, and then the cells were incubated with the fresh medium which contained various concentrations of ACP NPs or ANP NPs for 24 h. Subsequently, the cell samples were cultured with the fresh medium containing MTT for 4 h. Finally, the formazan crystal accumulated on the bottom was dissolved with dimethyl sulfoxide and the absorption at 490 nm of the cell samples was monitored by a microplate reader. The MTT results in **Figure S4** showed that the cell viabilities were higher than 98% when the concentrations of ACP NPs or ANP NPs ranged from 0.02 to 0.1 nM. Moreover, the cell viability remained at 93.7% when the concentration of nanoparticles reached 0.2 nM. These results demonstrated the ACP NPs and ANP NPs possessed outstanding biocompatibility.

### Cytokine Detection

To evaluate the maturation and activation of DC after co-incubation with the ACP NPs, the cell supernatant was collected for cytokine analysis. The cells co-incubated with ANP NPs or Au NPs and the culture medium was set as the control. Tumor necrosis factor α (TNF-α) and interleukin-6 (IL-6) were monitored by the cytokine-specific ELISA kits. As shown in **Figure S5**, the levels of TNF-α and IL-6 secreted by the cells that treated with ACP NPs were significantly higher than those treated with ANP NPs or Au NPs, especially when the incubation time extended to more than 8 h. The TNF-α and IL-6 in the supernatant without any treatment remained at a low level. These results illustrated that the DCs were mature after being treated with the ACP NPs, while the cells treated with ANP NPs or Au NPs were still immature.

### Flow Cytometry

To further evaluate the activation effect of ACP NPs on DC2.4 cells, flow cytometry was utilized to monitor the expression levels of CD80 and CD86 in DC2.4 cells co-incubated with ACP NPs or ANP NPs for 2 h. Cells without any treatment served as the control. As demonstrated in **Figure S6**, the expression levels of CD80 and CD86 in DC2.4 cells treated with ACP NPs were significantly higher than those in the ANP NPs or the control group. These results further confirmed the maturation of dendritic cells induced by ACP NPs.

### Raman Spectra Analysis

**Figure 2:**
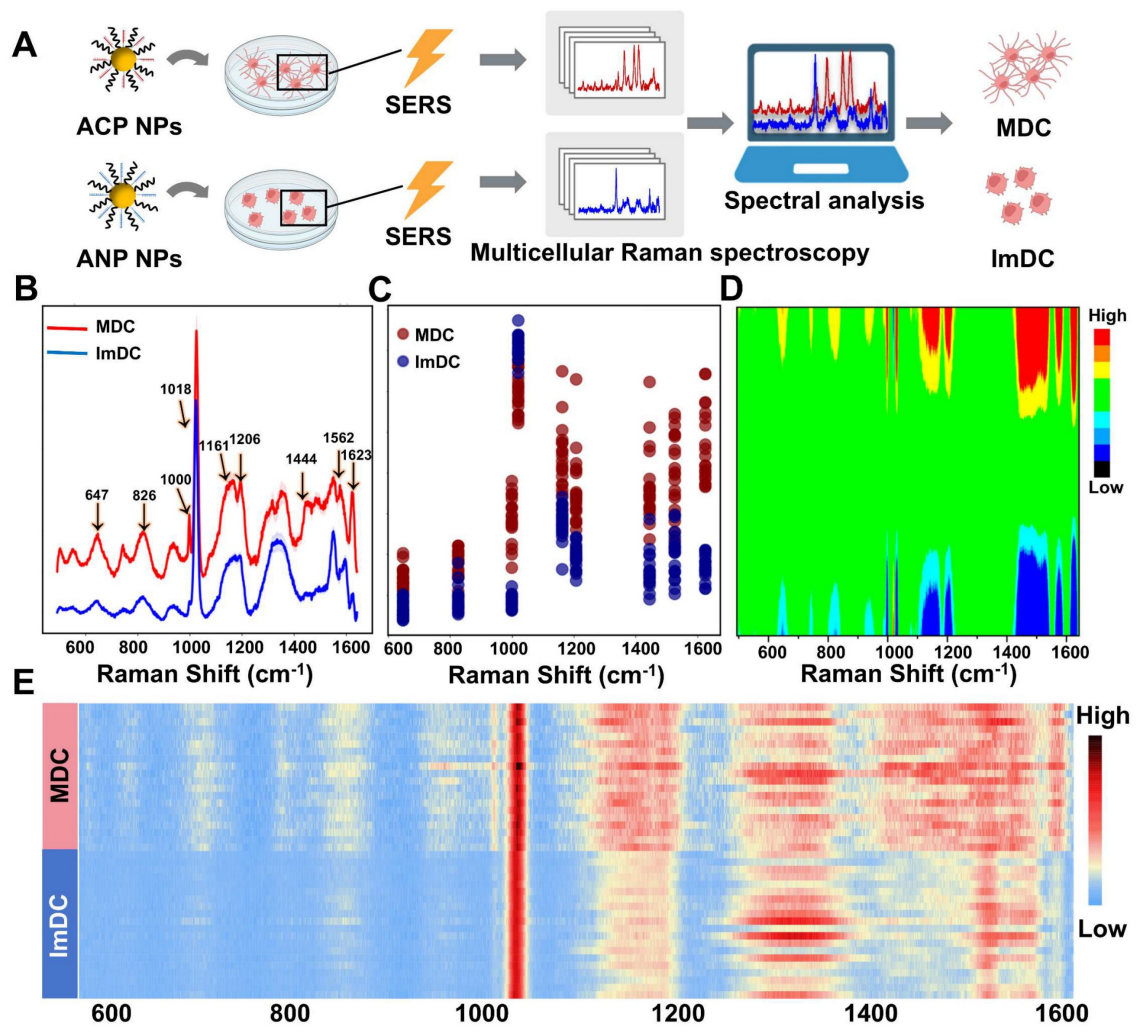
(A) Cell activation artificial intelligence process of the multiple DCs. (B) SERS spectra of DCs co-incubated with ACP NPs and ANP NPs with 95% confidence interval ranges. (C) Detailed display of the Raman intensity at 647, 826, 1000, 1018, 1161, 1206, 1444, 1562, and 1623cm^-1^ between the spectra. (D) Heatmap representation of Raman spectra (E) Multiple average Raman spectra randomly chosen from each cell type.

MDC displays significant variances in receptor expression, cytokine secretion, and nucleic acid profiles when contrasted with ImDC. Throughout the maturation process, discernible phenotypic markers of DCs emerge. To elucidate this process, we obtained the Raman fingerprints of MDC and ImDC, and subsequently discerned Raman spectral biomarkers associated with maturation. Raman spectroscopy offers unparalleled fingerprinting capabilities for biomolecules, facilitating the extraction of rich biological insights from cells. To investigate the disparities in Raman fingerprints between the mature and ImDC and identify maturation-associated spectral markers, the SERS spectra of MDC and ImDC were acquired and analyzed (**Figure 2A**). The averaged Raman spectra with a 95% confidence interval depicted in **Figure 2B** illustrate the typical signatures of each cell type encompassing distinctive bands. In detail, substantial variations were observed between the Raman spectra of the two types of cells across various regions, including methionine (647 cm^-1^), O-P-O stretch of DNA (826 cm^-1^), phenylalanine (1000 cm^-1^), Stretching C-O of ribose (1018 cm^-1^), C-C/C-N stretching of proteins (1161 cm^-1^), tyrosine (1206 cm^-1^), COO- (1562 cm^-1^), fatty acid (1444 cm^-1^), and tryptophan (1623 cm^-1^) (**Figure 2C-D**) 70, 71. To further illustrate the disparity in the Raman characteristics between MDC and ImDC, multiple average Raman spectra were randomly chosen from each cell type for visualizing the differences (**Figure 2E**). The heatmap representation of Raman spectra provides additional clarity regarding the notable distinctions between the Raman spectra of the two cell types. These results underscore the potential of cell-characterized Raman fingerprint spectra as an innovative platform for distinguishing mature stations of DCs.

### Deep Learning Algorithm

**Figure 3:**
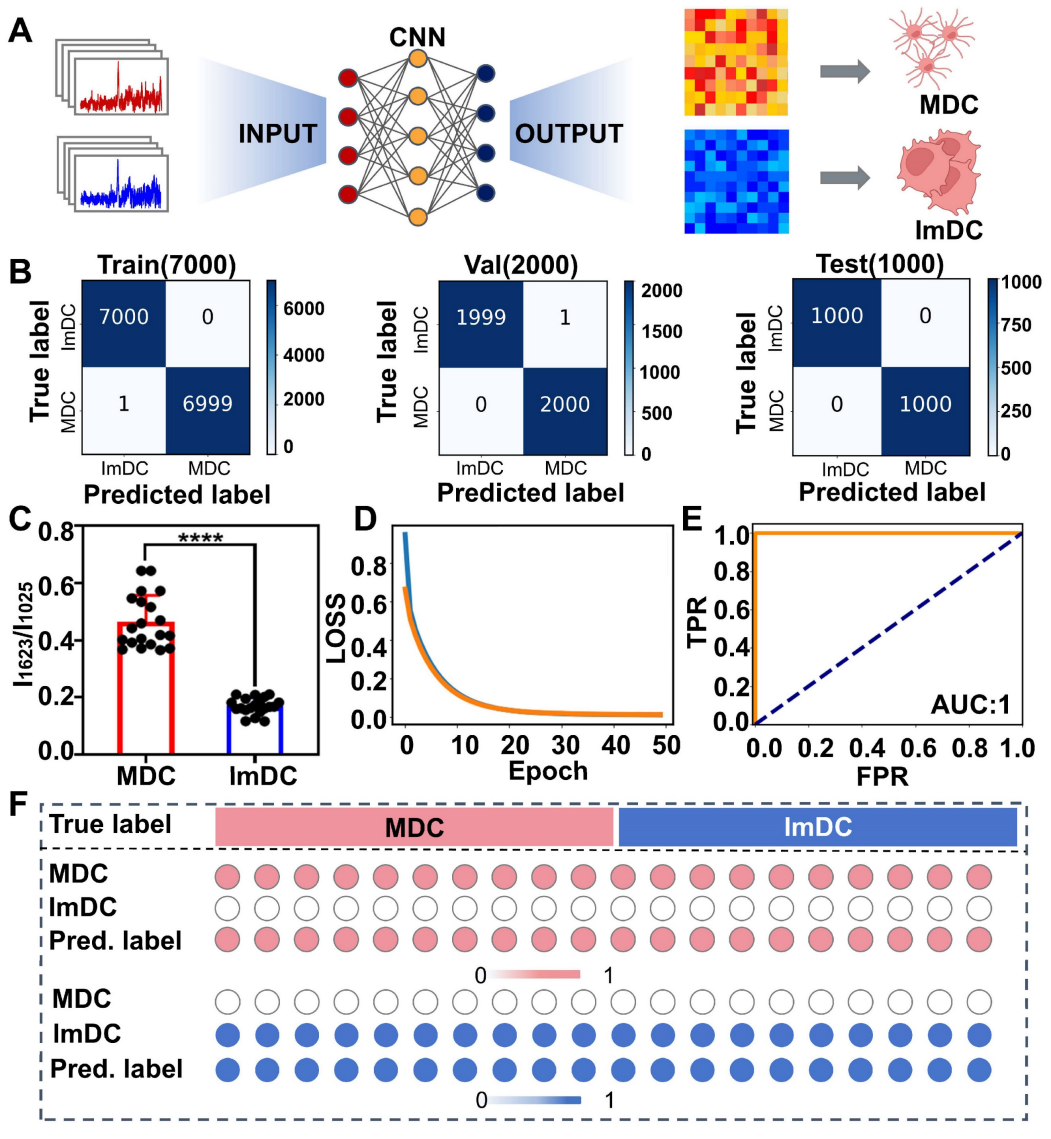
(A) Illustration of CNN model for DC maturation identification. (B) Confusion matrix for training, validation and test sets. (C) SERS intensity ratio of 1623 cm^-1^ to 1025 cm^-1^ from MDC and ImDC datasets (p < 0.0001). (D) LOSS curves. (E) ROC curves. (F) The output probabilities of each predicted cell present the classification accuracy of DC.

To identify the most suitable AI algorithm for spectral discrimination, we assessed the performance and theoretical advantages and disadvantages of three representative algorithms including CNN, Deep Neural Networks (DNN) and Multilayer Perceptron (MLP). Both CNN and DNN algorithms demonstrated superior accuracy (**Figure S7, Figure 3**). However, CNN could automatically extract spectral local features through convolutional layers, making it suitable for spectral data with complex patterns and overlapping peaks. Additionally, CNN exhibits good robustness against noise, enabling better handling of spectral noise that may occur during experiments. Ultimately, the CNN model was chosen for spectral classification. The CNN model conducts neural network computations through the utilization of filters and convolutional properties, has demonstrated notable success in image recognition tasks 72-76. Firstly, CNN can autonomously learn and extract valuable features from Raman spectra, thereby eliminating the need for manual feature extraction methods 77. This significantly streamlines data preprocessing, rendering it suitable for spectral analysis. Secondly, Raman spectra frequently display numerous overlapping peaks and intricate patterns, presenting significant challenges for conventional classification methods. The CNN algorithm adeptly addresses these complexities by leveraging the capabilities of the convolutional layers, offering robust classification capabilities 78, 79. Moreover, CNN demonstrates robustness against noise and minor spectral deformations by effectively learning local data features. After training, a CNN model can be easily deployed to rapidly classify large volumes of spectral data, facilitating efficient analysis and interpretation of complex datasets. The Raman spectra of MDC and ImDC were employed to train and validate the CNN-based binary classification model.

Firstly, the Conv1D layer consists of one-dimensional convolution (with 32 filters, kernel size=3, stride=1) and the ReLU activation function. The final output shape is (32, 1013, 32), Param=128. A dropout layer is typically added after the convolutional layer to prevent overfitting. This layer randomly sets a portion of the input units to 0 during training, aiding the model in learning more robust features. The MaxPooling 1D layer consists of a pooling layer, which is used to reduce the dimensionality and computational complexity of the data. With a pooling size of 2 (pool size=2) and a stride of 2, it takes the maximum value from every two consecutive data points as the output. The output shape is (32, 506, 32). The Flatten layer flattens multidimensional data into one-dimensional data so that it can be input into fully connected layers. The first Dense layer consists of a fully connected layer with 32 neurons. Similarly, it is equipped with the ReLU activation function. L2 regularization is added to prevent overfitting. The second Dense layer is the output layer of the model, consisting of only one neuron. The activation function is sigmoid, which is used to compress the model's output between 0 and 1, suitable for binary classification problems. A learning rate scheduler was added that reduces the learning rate when validation losses do not improve over several consecutive training cycles. This helps the model jump out of local optimality and continue to learn. Finally, the model is compiled with the binary cross-entropy loss function, which is also suitable for binary classification problems. The evaluation metric is accuracy, and the optimizer is stochastic gradient descent (SGD).

Compared to the default Adam optimizer, SGD updates the gradient for only one sample at a time, making it highly efficient in computation, especially when dealing with large-scale datasets. The CNN model has been customized to integrate convolutional kernels, pooling layers, and fully connected layers into one-dimensional modules tailored for processing Raman spectra. Moreover, the fully connected layers comprise two Dense layers: the first, equipped with 32 nodes and ReLU activation function, aids in further feature extraction; the second is an output layer with a single node and Sigmoid activation function, tailored for binary classification problems. The CNN model has been designed to ensure classification efficiency while mitigating the risk of overfitting and preserving simplicity in model structure and computational efficiency.

The CNN model excels in detecting subtle variations across the entire spectrum through neural network computations based on convolution features. By employing a filter, the correlation between spatially adjacent peaks in spectra is captured, enabling the extraction of abstracted spectral features through iterative filtering processes (**Figure 3A**). Initially, we randomly divided the Raman spectral data into training, validation, and testing sets, with proportions of 70%, 20%, and 10% respectively. The confusion matrix results demonstrated the trained models exhibited a commendable prediction accuracy, achieving 99.99% for the training dataset and 99.98% for the validation dataset (**Figure 3B**). The Raman intensity at 1623 cm^-1^ which is related to tryptophan, significantly contributed to the classification. By analyzing the weights in the final layer of the CNN, we quantified the contribution of each Raman peak to the model's classification performance (**Figure S8).** The results demonstrated that the peak at 1623 cm^-1^ possessed 6.1% contribution to the classification, making it the most significant among all the Raman peaks. Tryptophan catabolism is a known mechanism involved in immune system modulation and plays a multifaceted regulatory role in the antigen-presenting function of DCs and activation of T-cells, which is probably due to elevated indoleamine 2,3-dioxygenase (IDO) expression may reduce local tryptophan levels in ImDC 80, 81. Furthermore, the ratio of 1623 cm^-1^ to 1025 cm^-1^ (related to Carbohydrates) exhibited a clear distinction (P<0.0001), as shown in **Figure 3C**.

As the training iterations progressed, the discrepancy between model predictions and actual results gradually decreased, as depicted by the LOSS curve (**Figure 3D**). During the assessment of diagnostic values using a receiver operator curve (ROC), which demonstrated remarkable accuracy in classifying both training and validation samples, achieving an area under the curve (AUC) of 1.00 (**Figure 3E**). Additionally, we extracted weights from the final layer and monitored the predicted classification probabilities of the two cell types. Upon comparing heatmaps illustrating the classification outcomes, MDC and ImDC can be readily discerned (**Figure 3F**). These findings underscore the outstanding predictive accuracy of the CNN model for the maturity status of DCs.

**Figure 4:**
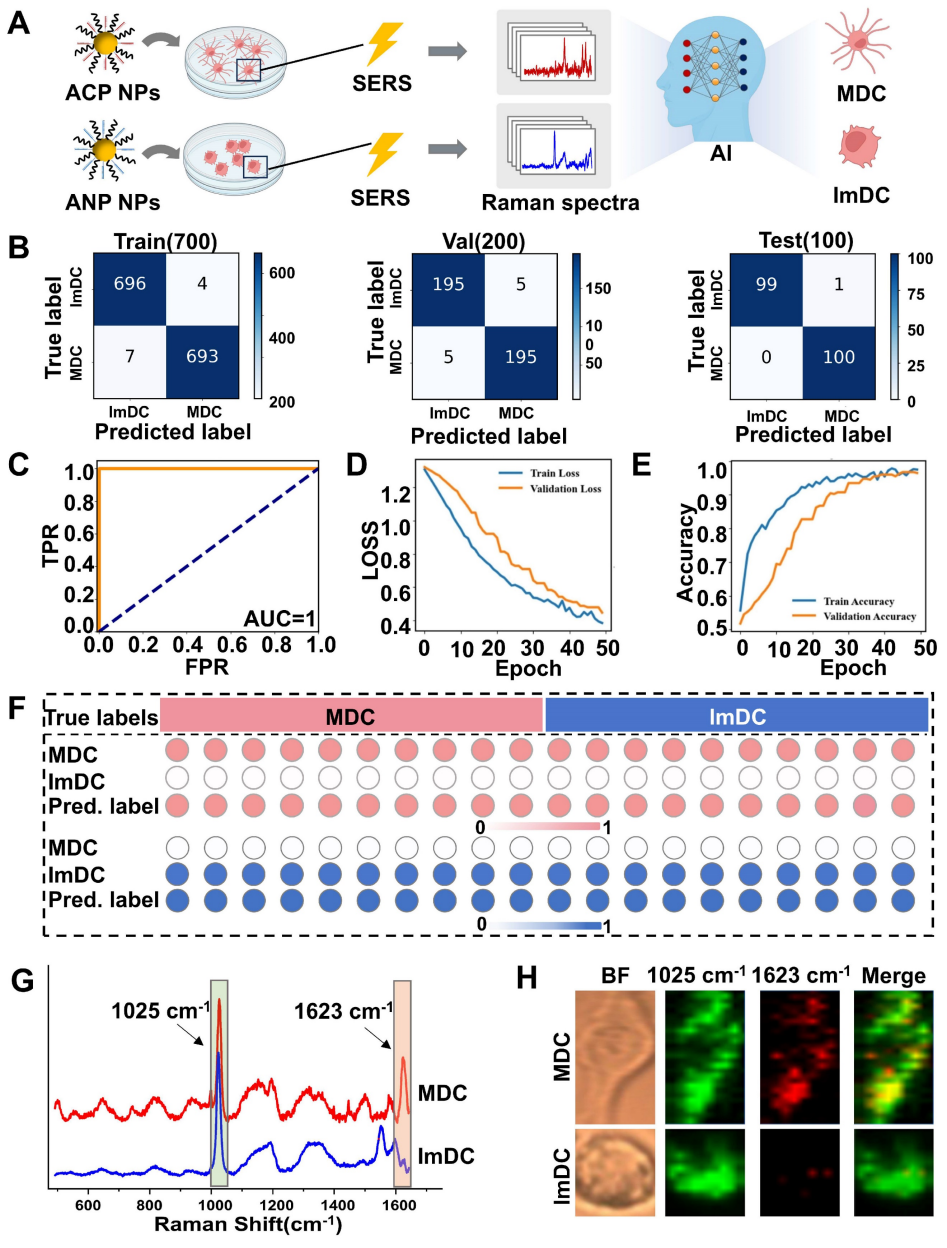
(A) Illustration of CNN model for individual DC maturation identification. (B) Confusion matrix representing the identification accuracy for each class of the proposed model. (C) ROC curves, (D) Loss curves and (E) accuracy curves. The loss and accuracy shown are the values recorded at the end of each training epoch. (F) The output probabilities of each predicted cell present the classification accuracy of single-DC. The top and bottom rows in each heatmap showed the true labels and predicted labels of the individual DC in each group. (G) Average Raman spectra of single MDC and ImDC. (H) Typical Raman mapping imaging of the two types of cells at the channel of 1025 cm^-1^ and 1623 cm^-1^.

Single cells represent the fundamental structural and functional units of all living organisms, integral to the functioning of multicellular systems. They exhibit considerable variability in molecular expression in response to external stimuli or pathological conditions, even among cells of the same type. Traditional analyses based on cell populations frequently mask these individual differences, leading to the loss of critical biological information. Single-cell analysis is not only a crucial approach for elucidating cellular heterogeneity and differentiation but also an effective strategy for precisely studying the relationships between biomolecules and associated signaling pathways 82, 83. The complex diversity of intracellular structures poses a challenge in extracting comprehensive information from individual cells, while most SERS research focuses on cell populations, overlooking the heterogeneity and diversity within individual cells. Delving into single-cell analysis becomes crucial for identifying distinct subgroups and uncovering intricate interactions among analytes. Supervised classification employing CNN was utilized to train classifier models using the spectral dataset and forecast the maturity status of the individual DC cells (**Figure 4A**). Around 20,000 Raman spectra were collected, derived from 196 mature DCs and 210 immature DCs. The average spectrum of individual cells was calculated and then randomly divided these average spectra into training, validation, and testing sets, each undergoing 50 epochs. Remarkably, the CNN model demonstrated strong performance in predicting the Raman spectra dataset, as evidenced by the results summarized in a confusion matrix. The sensitivity of the test set was 99% (**Figure 4B**).

Corresponding receiver operator curves (ROC) were plotted to illustrate classification performance, as shown in **Figure 4C**. The area under the curve (AUC) was 1.00. **Figures 4D and 4E** display the recognition loss and accuracy curves of the CNN model, showcasing the effective discriminative capacity. To gain deeper insights into the model's architecture and understand its output framework, we extracted weights from the final layer and then conducted statistical analysis on the output probabilities of each predicted mature or immature cell. We observed that almost every individual cell was accurately classified into either mature or immature categories from the heatmaps (**Figure 4F**). The precise prediction of the maturity status of DCs demonstrates that Raman spectra have the capability to capture intricate biomolecular characteristics of DCs, crucial in determining the maturation outcome.

As the significant discrepancy was observed of the intensity ratio of 1623 cm^-1^ to 1025 cm^-1^ in the Raman spectra of mature and immature DC cells, with mature cells displaying notably higher SERS intensity (**Figure 4G**). Raman mapping imaging of the two types of DCs was acquired at 1025 cm^-1^ and 1623 cm^-1^, respectively (**Figure 4H**). In the 1025 cm^-1^ channel, both mature and immature DC cells exhibited similar high intensity and abundant Raman signal distribution. However, in the 1623 cm^-1^ channel, the signal from MDC was significantly stronger, while almost imperceptible signals were detected in the immature cells. Under bright-field microscopy, mature cells displayed irregular elongated protrusions characteristic of mature DC morphology. The robust alignment between the CNN model predictions and the dynamic alterations observed in cellular Raman imaging implies the potential to differentiate MDC and ImDC at the single-cell level using deep learning-based SERS spectral analysis.

### Real-Time Identification of Dendritic Cell Maturation

**Figure 5:**
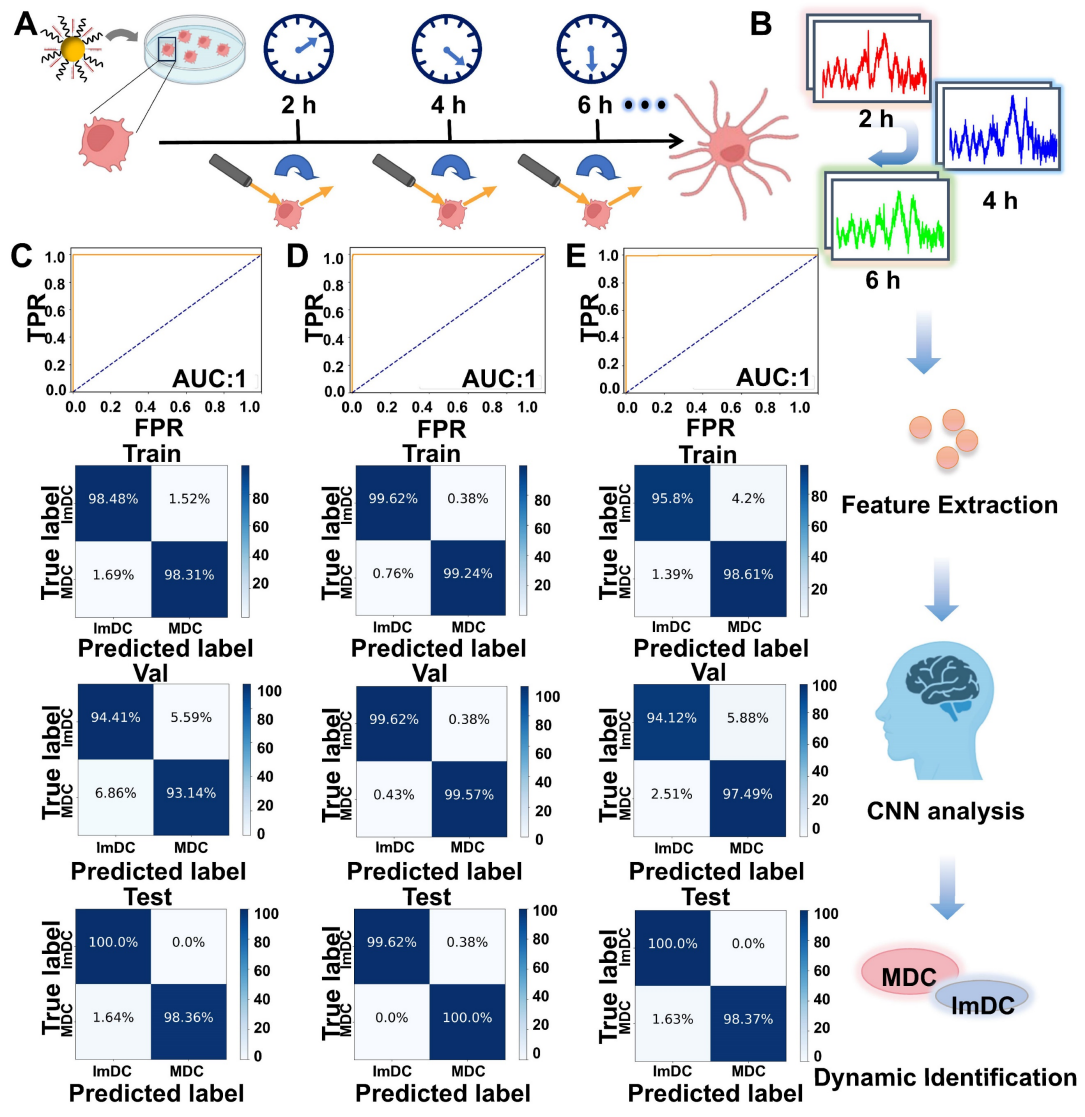
(A) Schematic diagram of real-time monitoring living DC maturation. (B) Monitoring the dynamic SERS spectra of living DCs at various time points and illustrating the schematic diagram of real-time dynamic classification of DC maturation through artificial intelligence. ROC curves and Confusion matrix for training, validation and test sets of SERS spectra collected from 2 h (C), 4 h (D) and 6 h (E).

To further validate the effectiveness of the deep learning-based SERS technique, we performed real-time SERS detection of individual living DCs at various time points (**Figure 5A**). SERS spectra of living DCs at various time points were dynamically monitored and the CNN model was used to real-time predict the maturity status of DC (**Figure 5B**). As evidenced by the results summarized in the confusion matrix, the classification accuracy was separately achieved 99.18%, 99.39%, 98.92% at various time points (2, 4 and 6 h). ROC curves (2 h, 4 h) demonstrated remarkable accuracy in classifying both training and test samples, achieving an area under the curve (AUC) of 1.00, whereas ROC curves (6 h) demonstrated a slightly lower accuracy of 0.99. The specificities at 2 h, 4 h, and 6 h separately reached 98.36%, 98.78%, 97.83% (**Figure 5C-E**). **Figure S9** displayed the recognition loss and accuracy curves of the real-time classification model, showcasing the effective discriminative capacity. These findings further underscore the superiority of the deep learning-based SERS technique in discerning the maturity status of DCs.

## Conclusion

In summary, we introduced a novel platform for assessing the maturation status of dendritic cells, integrating SERS with artificial intelligence technology. The platform employed Au@CpG@PEG NPs as self-reporting nanovaccines to assess DC activation and maturation state through a label-free SERS approach, relying on Au NPs as Raman-enhanced substrates. The fingerprint vibrational spectra of biological components in various states of DCs were collected and analyzed using CNN algorithms, facilitating the rapid and accurate identification of DC maturation, with an accuracy of up to 99%. Utilizing the self-reporting Au@CpG@PEG nanovaccine in combination of SERS with deep learning technology enabled precise assessment of individual DC maturation status. Furthermore, this approach allowed for real-time monitoring and evaluation throughout the maturation process of individual DCs, achieving an accuracy above 98.92%. This platform offers a powerful tool for classifying the maturation status of DCs, reducing redundant experimental procedures, lowering testing expenses, facilitating real-time monitoring, and enabling rapid and accurate detection. In addition, the SERS intensity ratio of 1623 cm^-1^ to 1025 cm^-1^ (tryptophan-to-carbohydrate) has been found as a potential marker for the maturation status of DC cells. These findings indicated the capability of the CNN-based SERS strategy for dynamically real-time monitoring and classifying the maturation status of dendritic cells. This method holds clinical translational potential in autologous DC transfusion therapy for cancer patients, providing a means to detect DC maturation status after *ex vivo* expansion.

## Supplementary Material

Supplementary figures.
