# Supplementary material for: Real-time monitoring of single dendritic cell maturation using deep learning-assisted surface-enhanced Raman spectroscopy
Source: Theranostics. 2024 Oct 14;14(17):6818–30. doi: 10.7150/thno.100298 (PMC11519801; doi:10.7150/thno.100298)
Supplement: Supplementary file 1 — Supplementary figures. [file thnov14p6818s1.pdf]

Supporting Information

Cai Zhang[1*✉], Mengling Wang[1*], Houde Wu[3*], Hongmei Gao[5✉], Pengyun Wu[4], Li Guo[3✉], Dingbin Liu[2✉]

1. Department of Radiology, Tianjin Medical University Cancer Institute and Hospital, National Clinical Research Center for Cancer, Tianjin's Clinical Research Center for Cancer, Key Laboratory of Cancer Immunology and Biotherapy, Tianjin 300060, China.
2. College of Chemistry, Research Center for Analytical Sciences, State Key Laboratory of Medicinal Chemical Biology, Tianjin Key Laboratory of Molecular Recognition and Biosensing, Nankai University, Tianjin 300071, China.
3. School of Medical Imaging, Tianjin Medical University, Tianjin 300203, China.
4. Department of Nuclear Medicine, Tianjin Medical University General Hospital, Tianjin, 300052, China.
5. Department of Intensive Care Unit, Key Laboratory for Critical Care Medicine of the Ministry of Health, Emergency Medicine Research Institute, Tianjin First Center Hospital, Nankai University, Tianjin 300192, China

* These authors contributed equally to this work and should be considered co-first authors.

✉Corresponding authors: Dingbin Liu (liudb@nankai.edu.cn); Cai Zhang (caizhang@tmu.edu.cn); Li Guo (yxgl@tmu.edu.cn); Hongmei Gao (ghm182@163.com)

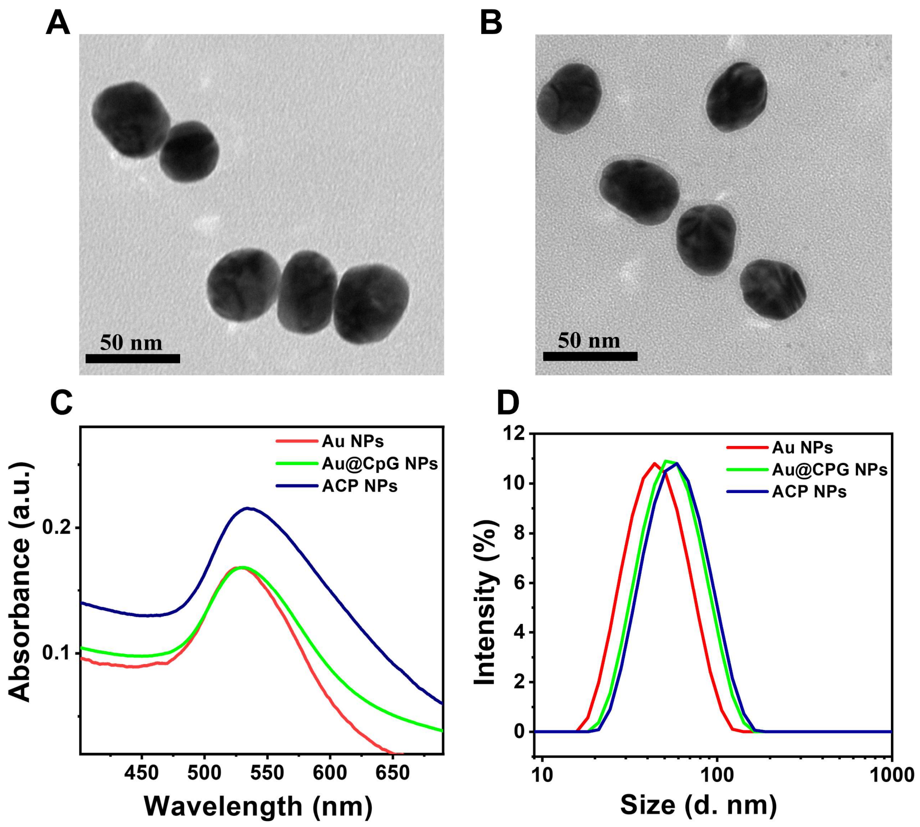

Figure S1. Characterization of the prepared nanoparticles. TEM images of Au NPs (A) and Au@CpG NPs (B). (C) UV-vis absorption spectra of Au NPs, Au@CpG NPs and ACP NPs. (D) Hydraulic diameters of the prepared nanoparticles.

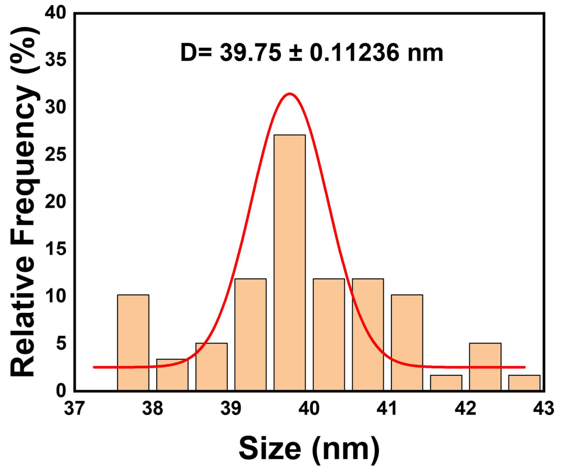

Figure S2. The size distribution histogram of Au NPs.

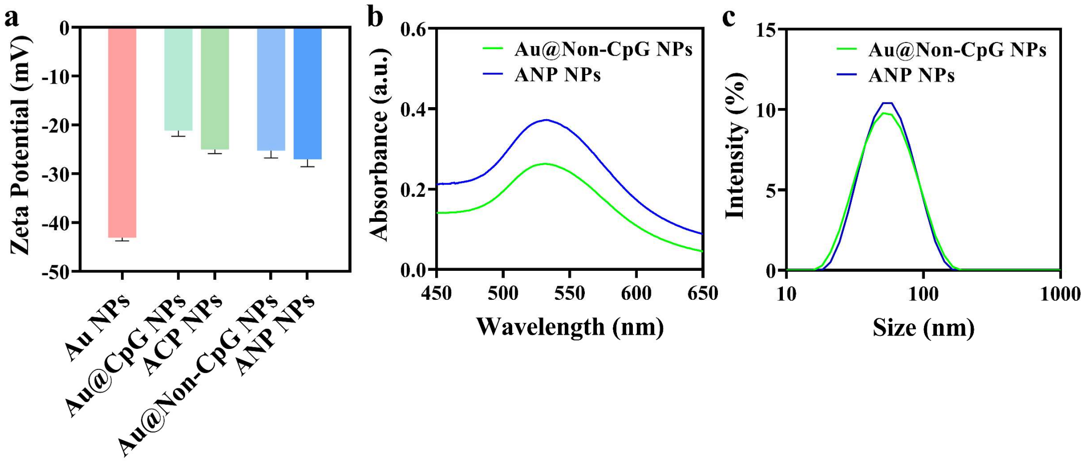

Figure S3. (A) Zeta potential of prepared nanoparticles. (B) UV-vis absorption spectra of Au@Non-CpG NPs and ANP NPs. (C) Hydraulic diameters of Au@Non-CpG NPs and ANP NPs.

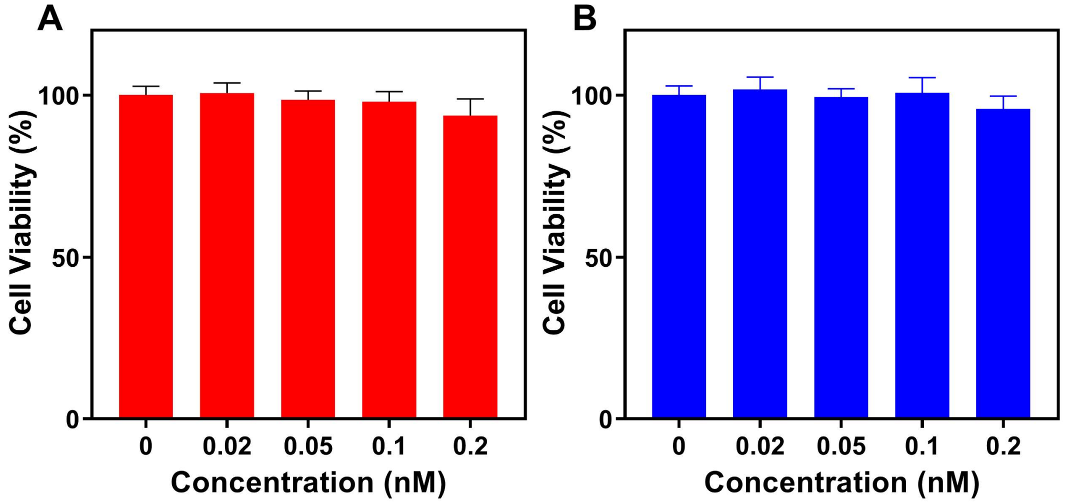

Figure S4. Cytotoxicity evaluation of the ACP NPs (A) and ANP NPs (B).

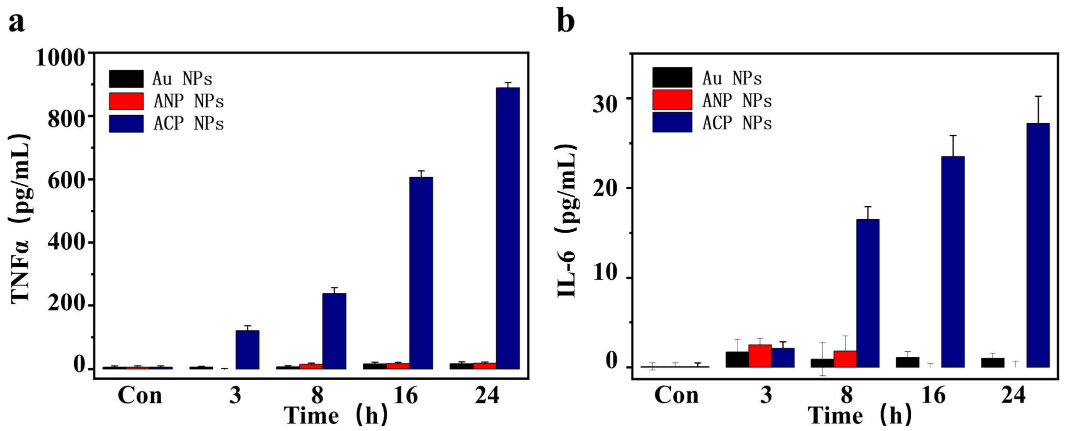

Figure S5. IL-6 (A) and TNF-α (B) detection of the DCs treated with Au NPs, ACP NPs or ANP NPs at different time points.

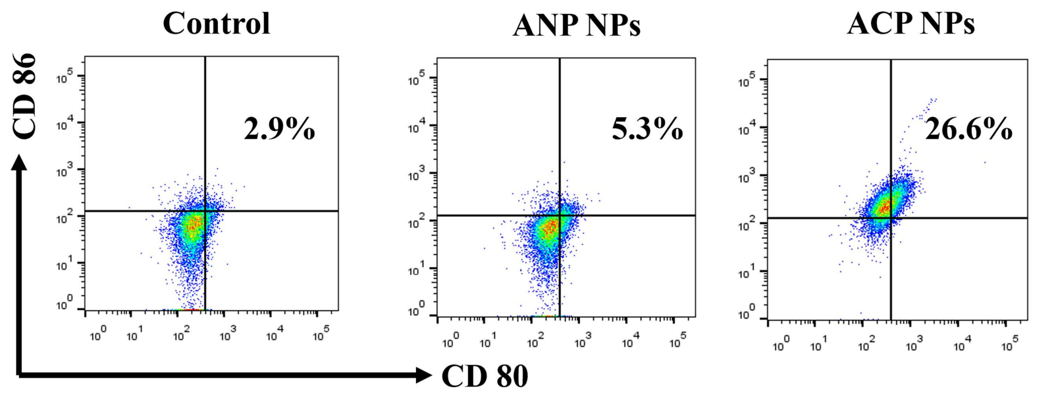

Figure S6. The expression levels of CD80 and CD86 markers in dendritic cells incubated with ANP or ACP nanoparticles, and the cell samples without any treatment were set as the control.

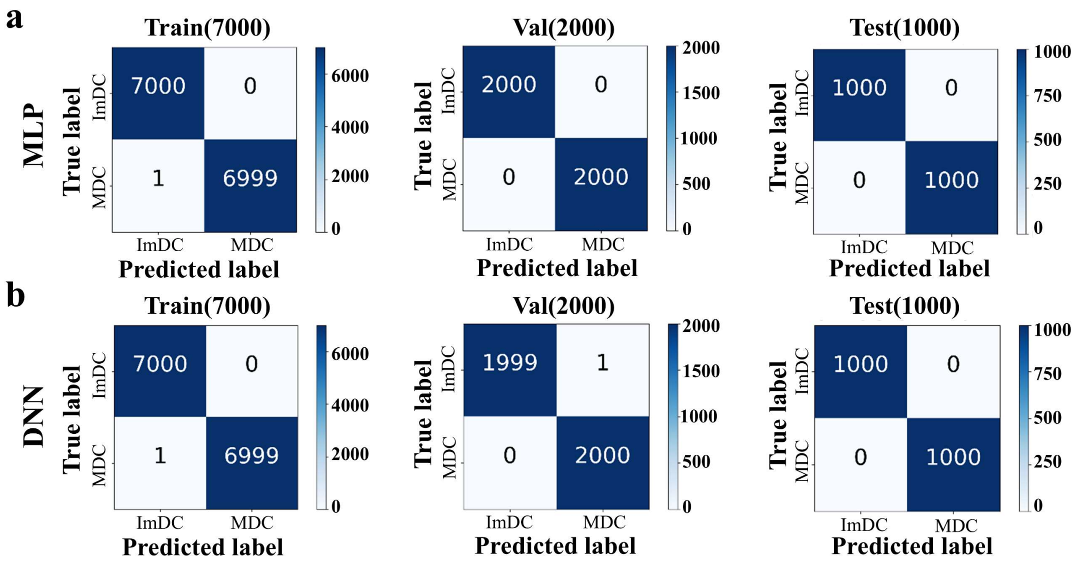

Figure S7. (A) Training, Validation and Test results from MLP algorithms. (B) Training, Validation and Test results from DNN algorithms.

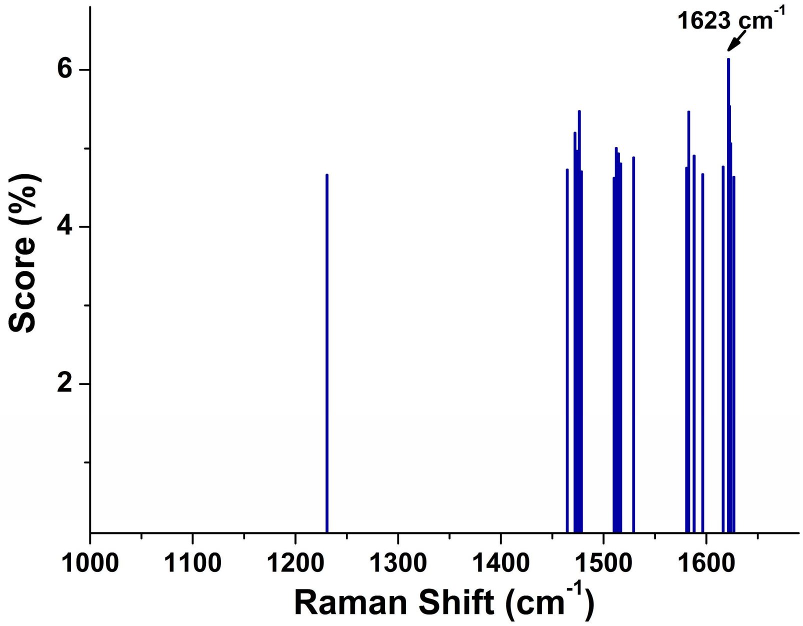

Figure S8. The contribution of individual Raman spectral peaks to the CNN model's classification performance.

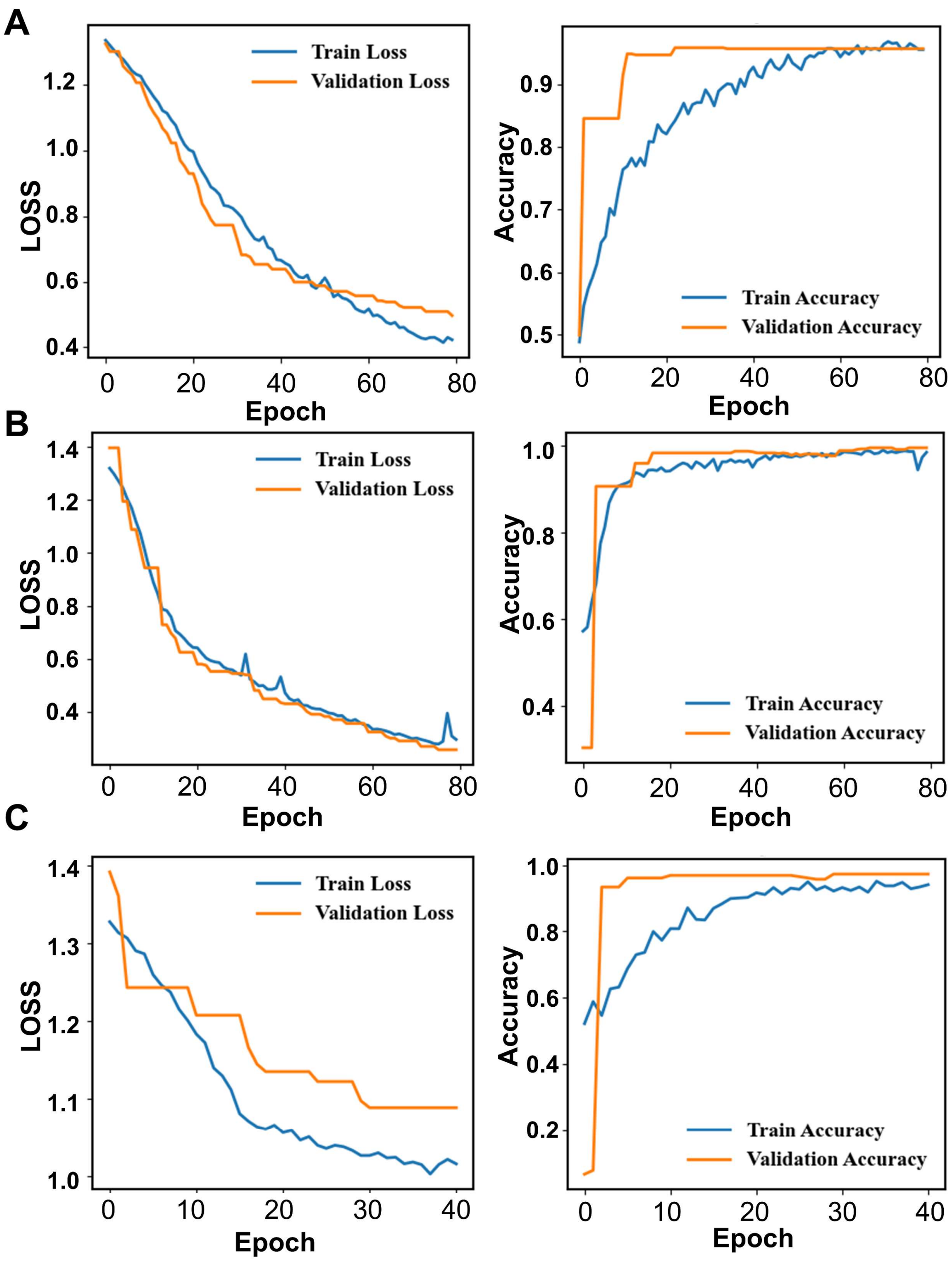

Figure S9. (A) LOSS and accuracy curves of the DCs status classification after incubation with nanoparticles for 2 h. (B) LOSS and accuracy curves of the DCs status classification after incubation with nanoparticles for 4 h. (C) LOSS and accuracy curves of the DCs status classification after incubation with nanoparticles for 6 h.
